# Trimethylamine N-Oxide, Circulating Endothelial Progenitor Cells, and Endothelial Function in Patients with Stable Angina

**DOI:** 10.1038/s41598-019-40638-y

**Published:** 2019-03-12

**Authors:** Ruey-Hsing Chou, Chi-Yu Chen, I-Chun Chen, Hsin-Lei Huang, Ya-Wen Lu, Chin-Sung Kuo, Chun-Chin Chang, Po-Hsun Huang, Jaw-Wen Chen, Shing-Jong Lin

**Affiliations:** 1 Institute of Clinical Medicine, National Yang-Ming University, Taipei, Taiwan; 2 Institute of Physiology, National Yang-Ming University, Taipei, Taiwan; 3 Cardiovascular Research Center, National Yang-Ming University, Taipei, Taiwan; 4 Institute and Department of Pharmacology, National Yang-Ming University, Taipei, Taiwan; 5 Division of Cardiology, Department of Medicine, Taipei Veterans General Hospital, Taipei, Taiwan; 6 Division of Endocrinology and Metabolism, Department of Medicine, Taipei Veterans General Hospital, Taipei, Taiwan; 7 Department of Critical Care Medicine, Taipei Veterans General Hospital, Taipei, Taiwan; 8 Healthcare and Management Center, Taipei Veterans General Hospital, Taipei, Taiwan; 9 Department of Medical Research, Taipei Veterans General Hospital, Taipei, Taiwan; 10 Department of Nursing, National Taipei University of Nursing and Health Sciences, Taipei, Taiwan; 11 Taipei Medical University, Taipei, Taiwan

## Abstract

Trimethylamine N-oxide (TMAO) is a metabolite originated from bacterial metabolism of choline-rich foods. Evidence suggests an association between TMAO and atherosclerosis, but the relationship between TMAO and endothelial progenitor cells (EPCs) remains unclear. This study aimed to identify the relationship between TMAO concentrations, circulating EPCs, and endothelial function in patients with stable angina. Eighty-one stable angina subjects who underwent coronary angiography were enrolled. The circulating EPCs and flow-mediated vasodilation (FMD) were measured to evaluate endothelial function. Plasma TMAO and inflammatory markers, such as hsCRP and IL-1β, were determined. Furthermore, the effect of TMAO on EPCs was assessed *in vitro*. Patients with lower FMD had significantly decreased circulating EPCs, elevated TMAO, hsCRP, and IL-1β concentrations. Plasma TMAO levels were negatively correlated with circulating EPC numbers and the FMD, and positively correlated with hsCRP, IL-1β concentrations. In *in vitro* studies, incubation of TMAO in cultured EPCs promoted cellular inflammation, elevated oxidative stress, and suppressed EPC functions. Enhanced plasma TMAO levels were associated with reduced circulating EPCs numbers, endothelial dysfunction, and more adverse cardiovascular events. These findings provided evidence of TMAO’s toxicity on EPCs, and delivered new insight into the mechanism of TMAO-mediated atherosclerosis, which could be derived from TMAO-downregulated EPC functions.

## Introduction

Due to improvements of bioinformatics technology, the role of intestinal microbiota in common diseases has been explored widely in recent years. Accumulating evidence indicates that the imbalance of intestinal microbiota is associated with coronary artery disease (CAD) and cardiovascular risk factors, including diabetes, dyslipidemia, obesity, and chronic kidney disease^1–3^. Trimethylamine N-oxide (TMAO) is a proinflammatory metabolite that originates from bacterial metabolism of choline-rich foods, such as red meats and eggs, in the large intestine and is rapidly oxidized by flavin-containing monooxygenase-3 in the liver^4^. TMAO has been found to contribute to vascular inflammation^5^, platelet hyperreactivity^6^, and atherosclerosis^7^. An elevated plasma TMAO level is an independent predictor of adverse cardiovascular events in the general population^8^ and CAD patients^9^. Dietary supplement with choline promote upregulation of macrophage receptors and enhance atherosclerosis in the apoE−/− mice. Deletion of gut microbiota by antibiotics cancels the proatherosclerotic effect and reduces plasma TMAO concentration at the same time^10^. In human umbilical vein endothelial cells (HUVECs), TMAO also has been suggested to be associated with endothelial dysfunction^5^, an early event in the development of atherosclerosis^11^. However, most studies indicating a relationship between TMAO and endothelial dysfunction are performed in rodents^12^ or cells^5^. Though Ke Y *et al*. had recently conducted a study to show the association between circulating TMAO and vascular aging in 186 subjects, suggesting TMAO may deteriorate the endothelial function by increasing oxidative stress in HUVECs, they did not provide clinical information about the endothelial functions of enrolled patients^13^. Clinical data interpreting the effect of TMAO on endothelial function are still very limited.

Many clinical and basic studies have provided evidence that endothelial progenitor cells (EPCs) play a crucial role in restoring endothelial injury and maintaining endothelial function. When vascular injury occurs, bone marrow-derived EPCs can mobilize to the peripheral circulation and differentiate into endothelial cells at sites of damaged endothelium^14^. The number of circulating EPCs also has been reported to correlate inversely with the risk factors for CAD^15^ and to be associated with peripheral^16^ and coronary endothelial function^17^. However, the relationship between circulating EPCs and TMAO concentration as well as the effects of TMAO on EPCs remain unclear. Therefore, we hypothesized that an enhanced TMAO level might provoke inflammation and induce changes in the numbers and function of EPCs, finally contributing to endothelial dysfunction. In this single-center translational study, we investigated the association between TMAO, circulating EPCs, and endothelial function in patients with stable angina. Also, to confirm the causal relationship between TMAO and endothelial dysfunction, we treated human EPCs with different concentrations of TMAO.

## Results

### Baseline characteristics and clinical outcomes

Totally 81 patients with stable angina and admitted for coronary angiography were enrolled for analysis. The median age of the study population was 68 years old, 69% of the subjects were male, and 62% of the subjects had significant CAD. Table 1 summarizes the baseline characteristics of the patients grouped by the FMD values. Patients with low FMD values had significantly lower eGFR levels, fewer circulating EPCs, and significantly higher levels of hsCRP, IL-1β, and TMAO.

**Table 1:** Baseline characteristics of patients with angina pectoris grouped by the values of flow-mediated dilation (FMD).

	Total (n = 81)	Low FMD (n = 40)	High FMD (n = 41)	*P*
Clinical variables				
Age (years)	68 (60–77)	64 (59–78)	72.0 (61–77)	0.688
Male gender, n (%)	56 (69)	26 (65)	30 (73)	0.477
Body mass index (kg/m^2^)	25 (23–28)	25 (23–27)	25 (24–28)	0.395
Smoking, n (%)	35 (43)	20 (50)	15 (37)	0.266
Hypertension, n (%)	52 (64)	30 (75)	22 (54)	0.064
Diabetes, n (%)	32 (40)	17 (43)	15 (37)	0.653
Heart failure, n (%)	12 (15)	8 (20)	4 (10)	0.226
Previous MI, n (%)	2 (3)	2 (5)	0 (0)	0.241
Previous stroke, n (%)	9 (11)	6 (15)	3 (7)	0.312
Hemoglobin (g/dL)	12.9 (11.5–14.2)	12.4 (11.4–14.1)	12.9 (11.8–14.3)	0.539
eGFR (mL/min/1.73 m^2^)	67.6 (52.9–76.6)	60.3 (44.7–73.0)	69.9 (58.6–80.0)	0.014
Fasting glucose (mg/dL)	99.0 (92.0–122.0)	100.5 (91.0–122.0)	98.0 (92.0–140.5)	0.704
Total cholesterol (mg/dL)	157.5 (138.3–176.0)	160.0 (135.5–172.8)	156.0 (145.8–189.0)	0.283
Triglyceride (mg/dL)	111.0 (87.0–132.8)	111.5 (91.8–144.8)	106.0 (86.0–128.5)	0.416
Uric acid (mg/dL)	6.1 (4.9–7.3)	6.1 (4.7–7.2)	6.3 (5.2–7.3)	0.470
Cardiac catheterization				
Significant CAD	51 (63)	27 (68)	24 (59)	0.492
SYNTAX score	6.5 (0.0–15.0)	7.0 (0.0–20.0)	4.0 (0.0–14.8)	0.227
LVEDP (mmHg)	22.0 (18.0–27.0)	22.0 (19.0–27.3)	22.0 (16.0–27.0)	0.686
LV ejection fraction (%)	56.6 (52.5–61.0)	56.6 (52.3–59.8)	58.0 (53.0–62.5)	0.432
Inflammatory biomarkers				
hsCRP (mg/dL)	0.2 (0.1–0.3)	0.3 (0.2–0.5)	0.1 (0.0–0.2)	<0.001
IL-1β (pg/mL)	0.2 (0.1–0.2)	0.2 (0.2–0.3)	0.1 (0.1–0.2)	<0.001
TMAO (μmol/L)	3.3 (2.3–7.6)	5.4 (2.4–13.2)	3.2 (2.1–4.1)	0.014
Endothelial functions				
Endothelial progenitor cells				
CD34^+^, KDR^+^ (%)	0.7 (0.5–0.9)	0.6 (0.4–0.7)	0.7 (0.5–1.4)	0.018
CD34^+^, KDR^+^, CD133^+^ (%)	0.6 (0.5–0.9)	0.6 (0.4–0.7)	0.7 (0.5–1.3)	0.028
Flow-mediated dilation (%)	7.0 (4.5–10.2)	4.5 (3.0–5.1)	9.5 (8.4–16.5)	<0.001

MI, myocardial infarction; eGFR, estimated glomerular filtration rate; CAD, coronary artery disease; LVEDP, left ventricular end-diastolic pressure; LV, left ventricle; hsCRP, high-sensitivity C-reactive protein; IL-1β, interkeukin-1β; KDR, kinase-insert domain-containing receptor.

**Figure 1:**
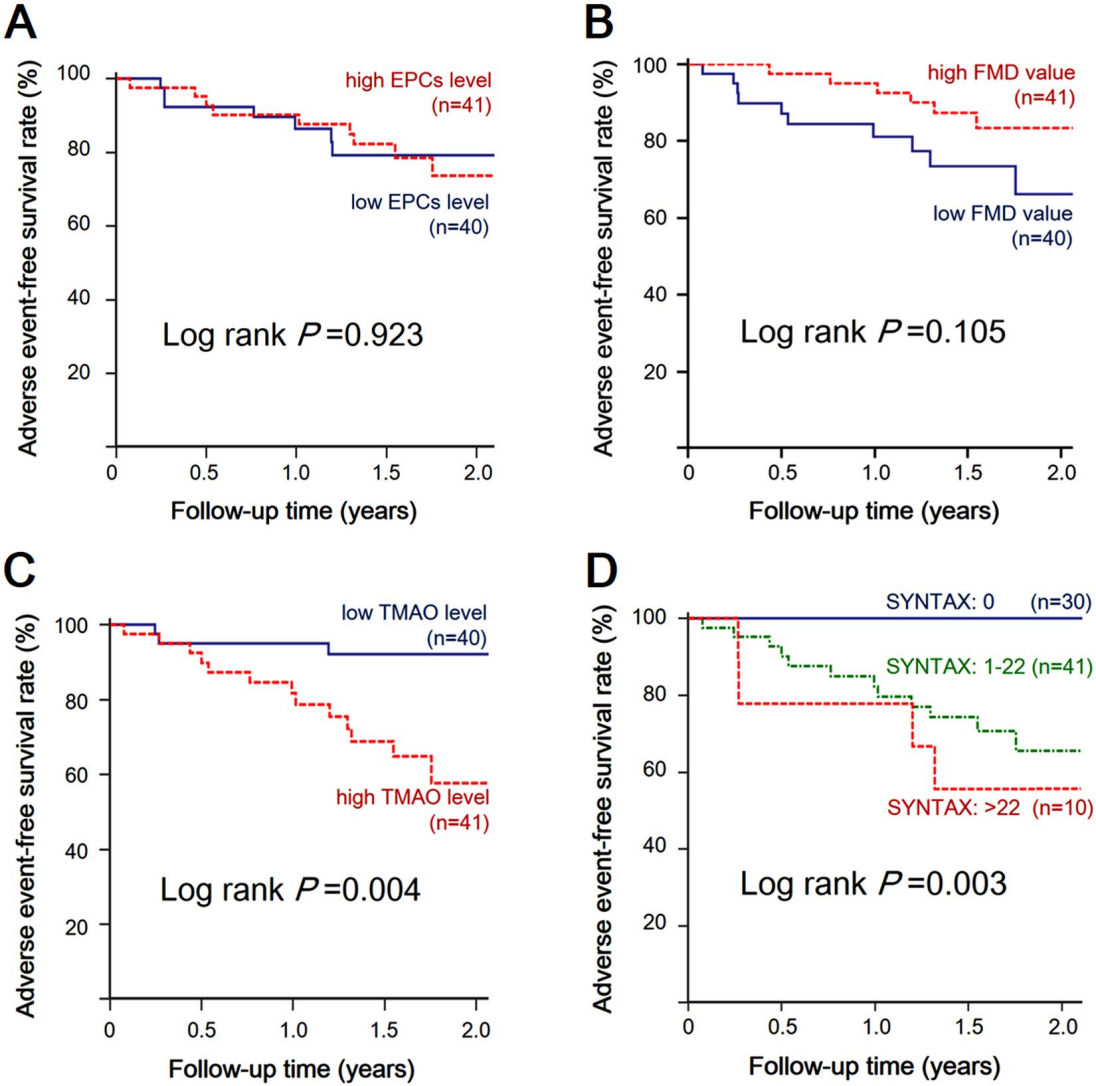
Kaplan–Meier curves of freedom from major adverse cardiovascular events in patients grouped by **(A)** circulating endothelial progenitor cells (EPCs), **(B)** flow-mediated dilation (FMD) values, **(C)** trimethylamine N-oxide (TMAO) concentrations, and **(D)** SYNTAX scores.

During the median follow-up duration of 1.5 years (range, 1.1–1.9 years), 16 cases (19.8%) of MACEs occurred after CAG, including 14 cases (17.3%) of target vessel revascularization and 2 cases (2.5%) of nonfatal MI. Kaplan–Meier survival analysis was performed to investigate the impact of endothelial function, TMAO, and SYNTAX score on adverse event-free survival, as illustrated in Fig. 1. Patients with low FMD tended to have a higher incidence of MACEs (10 cases, including 9 cases of target vessel revascularization and 1 case of MI), but the difference between the two groups was not statistically significant (log rank *p* = 0.105). Similarly, patients with lower circulating EPC levels were not associated with an increased occurrence of MACEs (log rank *p* = 0.923). Conversely, patients with either a higher TMAO concentration (log rank *p* = 0.004) or a higher SYNTAX score (log rank *p* = 0.003) were both significantly associated with an increased incidence of MACEs.

### Relationship between TMAO and endothelial function, EPCs, and inflammation

The correlation between TMAO, FMD, and various clinical factors are summarized in Table 2. The plasma TMAO concentration was positively correlated with the SYNTAX score (R = 0.244, *p* = 0.034), hsCRP (R = 0.276, *p* = 0.013), and IL-1β (R = 0.332, *p* = 0.003); but was negatively correlated with eGFR (R = −0.279, *p* = 0.012), circulating EPC (R = −0.260, *p* = 0.019 for CD34^+^KDR^+^ cells; R = −0.245, *p* = 0.028 for CD34^+^KDR^+^CD133^+^ cells), and FMD (R = −0.377, *p* = 0.001). The endothelium-dependent FMD values were positively correlated with circulating EPCs (R = 0.307, *p* = 0.005 for CD34^+^KDR^+^ cells; R = 0.275, *p* = 0.013 for CD34^+^KDR^+^CD133^+^ cells) and were negatively correlated with the concentrations of inflammatory cytokines (R = −0.450, *p* < 0.001 for hsCRP; R = −0.550, *p* < 0.001 for IL-1β) and TMAO (R = −0.377, *p* = 0.001).

**Table 2:** Correlation coefficients of circulating trimethylamine N-oxide (TMAO) concentration, flow-mediated dilation (FMD), and various clinical variables.

Variables	TMAO		FMD	
	R	*P* value	R	*P* value
Clinical variables				
Age (years)	0.044	0.693	0.117	0.297
Body mass index (kg/m^2^)	−0.035	0.756	0.028	0.804
Hemoglobin (g/dL)	−0.210	0.059	0.021	0.852
eGFR (mL/min/1.73 m^2^)	−0.279	0.012	0.284	0.011
Fasting glucose (mg/dL)	0.177	0.126	−0.007	0.954
Total cholesterol (mg/dL)	−0.166	0.141	0.153	0.175
Triglyceride (mg/dL)	0.198	0.078	−0.084	0.457
Uric acid (mg/dL)	0.004	0.972	0.067	0.555
Cardiac catheterization				
SYNTAX score	0.244	0.034	−0.145	0.211
LVEDP (mmHg)	0.059	0.636	−0.146	0.238
LVEF (%)	−0.207	0.063	0.123	0.272
Inflammatory biomarkers				
hsCRP (mg/dL)	0.276	0.013	−0.450	<0.001
IL-1β (pg/mL)	0.332	0.003	−0.550	<0.001
TMAO (μmol/L)	—	—	−0.377	0.001
Endothelial functions				
CD34^+^, KDR^+^ cells (%)	−0.260	0.019	0.307	0.005
CD34^+^, KDR^+^, CD133^+^ cells (%)	−0.245	0.028	0.275	0.013
Flow-mediated dilation (%)	−0.377	0.001	—	—

eGFR, estimated glomerular filtration rate; LVEDP, left ventricular end-diastolic pressure; LVEF, left ventricular ejection fraction; hsCRP, high-sensitivity C-reactive protein; IL-1β, interkeukin-1β; KDR, kinase-insert domain-containing receptor.

According to univariate linear regression analysis, hypertension, heart failure, decreased eGFR, circulating EPCs (both CD34^+^KDR^+^ and CD34^+^KDR^+^CD133^+^ cells), and increased inflammatory biomarkers (hsCRP, IL-1β, and TMAO) were all significantly associated with lower FMD. According to multivariate regression analysis, circulating EPCs (Std β = 0.291, *p* = 0.001 for CD34^+^KDR^+^ cells), IL-1β (Std β = −0.451, *p* < 0.001), and TMAO (Std β = −0.212, *p* = 0.040) remained significantly associated with the FMD levels, as shown in Table 3. Decreased circulating EPCs and increased inflammatory biomarkers, including IL-1β and TMAO, were independent risk factors of endothelial dysfunction.

**Table 3:** Univariate and multivariate linear regression analyses of factors associated with flow-mediated dilation (FMD)*.

Variables	Univariate analysis		Multivariate analysis^†^	
	Std β	*P* value	Std β	*P* value
Clinical variables				
Age (years)	0.101	0.370		
Male gender	0.022	0.845		
Body mass index (kg/m^2^)	0.059	0.600		
Smoking	−0.092	0.413		
Hypertension	−0.245	0.027		
Diabetes	−0.218	0.051		
Heart failure	−0.253	0.022		
Previous MI	−0.037	0.741		
Previous stroke	−0.062	0.583		
Hemoglobin (g/dL)	0.015	0.897		
eGFR (mL/min/1.73 m^2^)	0.309	0.005		
Fasting glucose (mg/dL)	−0.097	0.402		
Total cholesterol (mg/dL)	0.111	0.327		
Triglyceride (mg/dL)	−0.042	0.711		
Uric acid (mg/dL)	−0.002	0.984		
Cardiac catheterization				
Significant CAD	0.013	0.909		
SYNTAX score	−0.183	0.113		
LVEDP (mmHg)	−0.185	0.134		
LVEF (%)	0.173	0.122		
Inflammatory biomarkers				
hsCRP (mg/dL)	−0.325	0.003		
IL-1β (pg/mL)	−0.579	<0.001	−0.451	<0.001
TMAO (μmol/L)	−0.505	<0.001	−0.212	0.040
Endothelial progenitor cells				
CD34^+^, KDR^+^ (%)	0.387	<0.001	0.291	0.001
CD34^+^, KDR^+^, CD133^+^ (%)	0356	<0.001		

*Log transformation was performed to achieve normality before analysis.

^†^The model consisted of variables with *p* value < 0.1 in the univariate comparative test.

MI, myocardial infarction; eGFR, estimated glomerular filtration rate; CAD, coronary artery disease; LVEDP, left ventricular end-diastolic pressure; LVEF, left ventricular ejection fraction; hsCRP, high-sensitivity C-reactive protein; IL-1β, interkeukin-1β; TMAO, trimethylamine N-oxide; KDR, kinase-insert domain-containing receptor.

### Effects of TMAO on human EPCs

**Figure 2:**
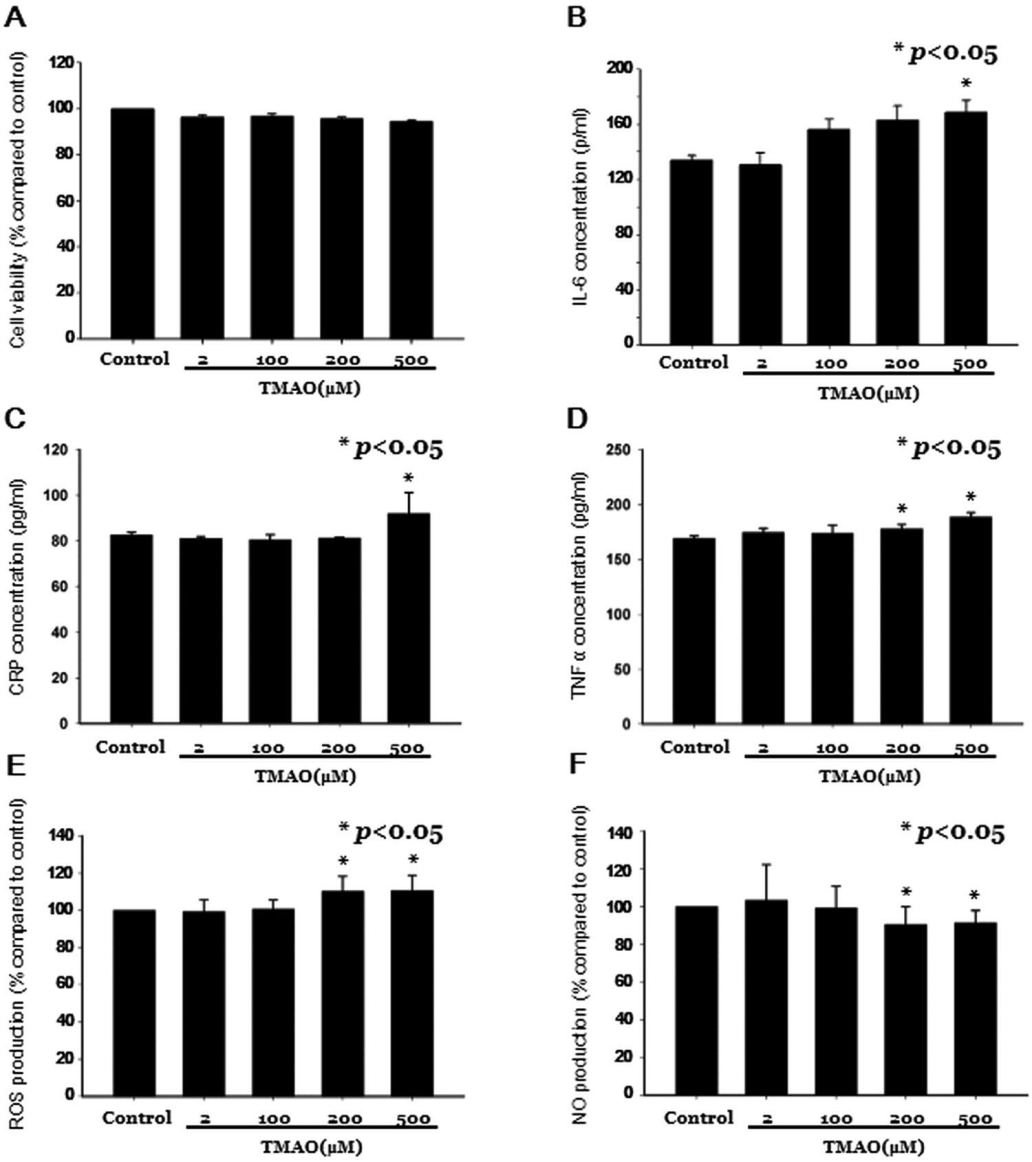
*In vitro* study investigating the impact of trimethylamine N-oxide (TMAO) on endothelial progenitor cells (EPCs). Human EPCs were cultured, the **(A)** cell viability and the contents of intracellular **(B)** interleukin-6 (IL-6), **(C)** C-reactive protein (CRP), **(D)** tumor necrosis factor-α (TNF-α), **(E)** reactive oxygen species (ROS) production, and **(F)** nitric oxide (NO) production were measured under different TMAO concentrations (0, 2, 100, 200, and 500 μM).

**Figure 3:**
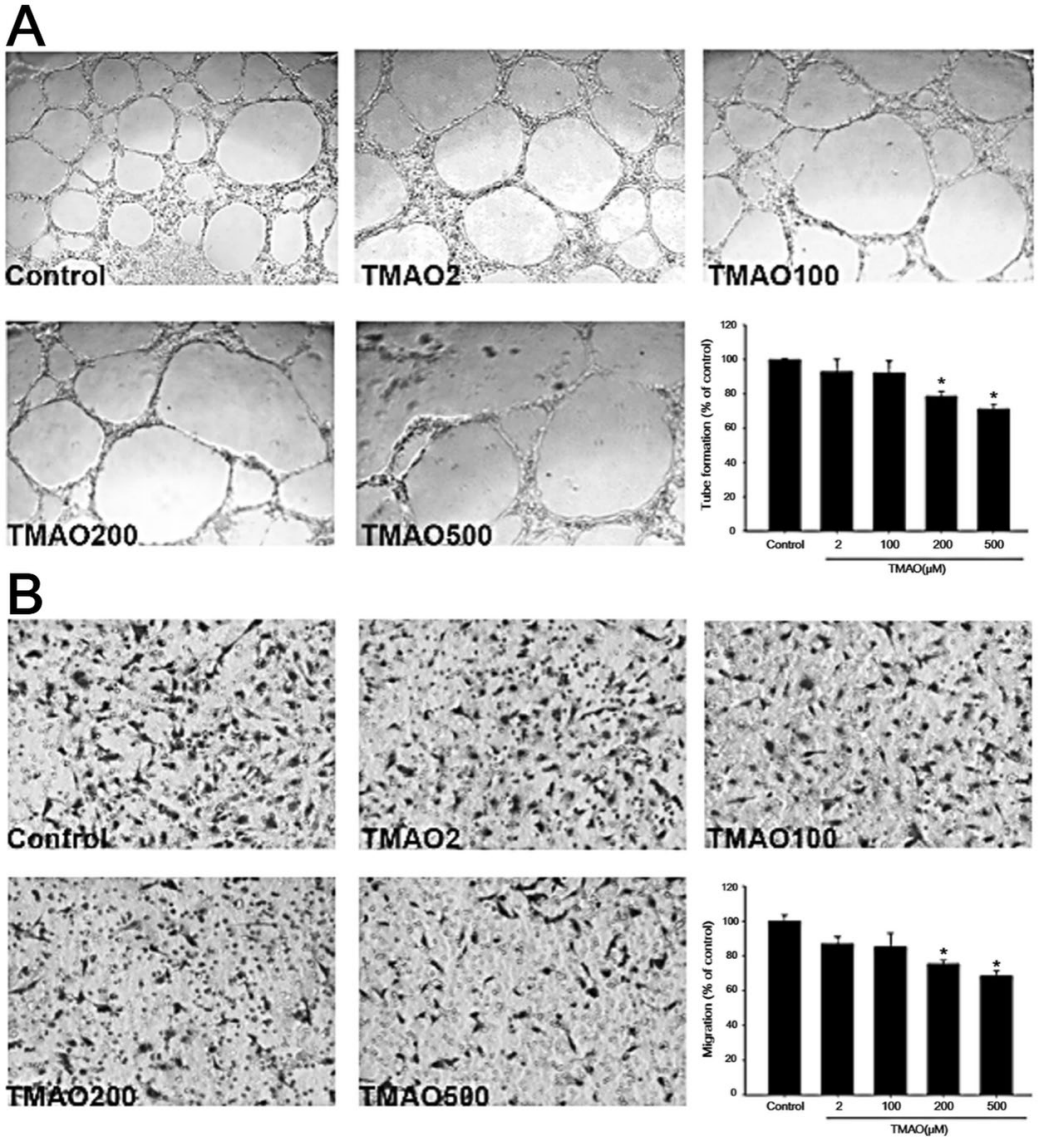
*In vitro* study investigating the impact of trimethylamine N-oxide (TMAO) on the functions of endothelial progenitor cells (EPCs). Human EPCs were cultured. Its abilities of **(A)** tube formation and **(B)** migration were measured under different TMAO concentrations (0, 2, 100, 200, and 500 μM).

We further tested the potential effects of TMAO on cultured EPCs in *in vitro* studies. The cell viability of human EPCs was not significantly different after incubation of different concentrations TMAO for 24 hours (Fig. 2A). However, EPCs treated with high concentrations TMAO were found to have significant higher interleukin-6 (IL-6), CRP, and TNF-α concentrations (Fig. 2B–D**)**. TMAO treatment also stimulated ROS production and downregulated NO production in human EPCs (n = 3, in triplicate; Fig. 2E,F). Since circulating EPCs contributed to neovascularization by migration, proliferation, and capillary tube formation^18^, we further detected EPC’s abilities of tube formation and migration under different concentrations of TMAO. The functions of EPCs, including the abilities of tube formation (Fig. 3A) and migration (Fig. 3B), were all significantly impaired under high-dose TMAO treatment.

## Discussion

To our knowledge, this is the first study to clarify the relationship between TMAO and EPCs in humans. The significant findings of this study are that an elevated level of TMAO, a gut microbiota-dependent metabolite, is associated with increased inflammation, fewer circulating EPCs, and endothelial dysfunction in patients with stable angina. The plasma TMAO concentration was independently and negatively correlated with the level of circulating EPCs and the FMD values. Patients with higher TMAO levels were associated with an increased incidence of MACEs. In the *in vitro* studies, the TMAO-treated EPCs showed upregulated IL-6, CRP, TNF-α, and ROS as well as downregulated NO production. High-dose TMAO impaired the functions of EPCs. These findings provide evidence of the involvement of TMAO in vascular inflammation and endothelial dysfunction through downregulation of EPCs.

It is increasingly recognized that the intestinal microbiome has significant metabolic effects and maintains a delicate balance within the human host. TMAO is an intestinal microbiota-generated, proinflammatory metabolite, which has been demonstrated to be associated with atherosclerosis and various cardiovascular risk factors^10^. Endothelial dysfunction, characterized by a reduction of NO bioavailability, is an early sign of atherosclerosis development. Endothelial cells are switched from an NO-mediated, quiescent status toward activation by redox signaling^19^, leading to inflammation, vasoconstriction, and vascular remodeling^11^. Several animal and cell-based studies have revealed a link between TMAO and ROS, the principal element contributing to endothelial dysfunction. In HUVECs, TMAO stimulation increased intracellular ROS production and activated the thioredoxin-interacting protein (TXNIP)-nod-like receptor family pyrin domain containing 3 (NLRP3) pathway, whereas inflammatory cytokines were released in a dose- and time-dependent manner^5^. Similarly, the activation of NLRP3 inflammasomes by TMAO had been reported to increase cell permeability in the mouse carotid artery endothelial cells^20^. Besides, TMAO has been found to promote monocyte adherence and impair the self-repair capacity of human endothelial cells^21^. In an animal study, TMAO was shown to accelerate vascular inflammation and impair endothelium-dependent relaxation of the aorta in aged rats, which was restored by 3,3-dimethyl-1-butanol (an inhibitor of trimethylamine formation)^12^. Our study suggests that the plasma TMAO level is associated with endothelium-dependent FMD in patients with symptoms of angina. Increased plasma TMAO levels and fewer circulating EPCs were independent predictors of reduced FMD. This is the first clinical study to clarify the relationship between TMAO and circulating EPCs in humans, thus providing novel insights into the role of TMAO in endothelial dysfunction.

EPCs derived from bone marrow can mobilize to the peripheral circulation to regenerate the injured endothelium, playing an important role in maintaining endothelial function^14^. The inverse correlation between the plasma TMAO level and circulating EPCs found in the current study can be explained as follows. As a proinflammatory metabolite, the TMAO concentration is markedly increased in atherosclerotic diseases, including diabetes, chronic kidney disease, heart failure, and CAD^2^, which are also risk factors for fewer EPCs^14^. Shared risk factors may affect TMAO and EPCs simultaneously, leading to the reverse epidemiology, but cannot fully explain the results of the cell-based studies. Though there is currently no paper to investigate the mechanism about how TMAO influence the EPCs, just as what we observed in the study of HUVECs^5^, TMAO may increase intracellular ROS production and activate the NLRP3-TXNIP pathway, lead to the release of inflammatory cytokines (mainly IL-1β and IL-18)^5^, which have been demonstrated to be toxic to EPCs at high-dose exposure^22^. TMAO also inhibited the mRNA and protein expression of endothelial nitric oxide synthase and reduced NO production in HUVECs, which could be reversed by N-acetylcysteine treatment, a ROS inhibitor^5^. EPCs incubated with recombinant CRP exhibited decreased survival, differentiation, and angiogenic functions^23^. Furthermore, an enhanced TNF-α concentration contributes to fewer EPCs^24^. Incubation of EPCs with H_2_O_2_ had been reported to profoundly reduce the numbers of EPCs by inducing apoptosis^25^. In the presented study, cell viability was not significantly decreased under high concentrations of TMAO. Maybe 24 hours was not long enough to observe the cytotoxicity of TMAO on EPCs. Nevertheless, TMAO was found to enhance intracellular inflammation and ROS production, which directly causing functional impairment of circulating EPCs^26^.

Our findings support the hypothesis that an elevated TMAO level, via provoking intracellular inflammation and oxidative stress, may inhibit the functions of circulating EPCs, and finally lead to endothelial dysfunction. Though low-grade inflammation has been reported to induce EPC mobilization^27^, the increased numbers of EPCs in response to inflammatory stimulation may be functionally impaired^28^. The *in vitro* study showed reduced NO expression in TMAO-treated EPCs, suggesting that TMAO can directly impair the NO-mediated endothelial function even when the numbers of EPCs were not significantly decreased. So, it is not surprising that patients with fewer circulating EPCs were not associated with an increased occurrence of MACEs. However, the MACE incidence between patients with high and low FMD values did not achieve statistical significance in the present study. Compared to circulating EPCs or FMD, the severity of CAD and vascular inflammation seem to be more important prognostic factors for patients with angina pectoris.

Several limitations of this study should be mentioned. First, this is a single-center study with small case numbers, which may be insufficient to reveal the clinical impacts of EPCs and FMD. Patients with lower FMD levels trended to have higher prevalence of hypertension, heart failure, and higher incidence of MACE during the follow-up period. But none of above findings achieved statistical significance. The clinical significance of endothelial function should be investigated in a large-scale study. Second, the study cohort was composed of patients with relatively low severity of CAD. Only 37% of the cases had angiographically significant CAD, which may underestimate the inflammation-related injuries of TMAO and atherosclerosis. In addition, mean age of the enrolled subjects were relatively elder in the presented study, which may limit the generalization of our results. For example, our study did not found a significant correlation between age and plasma TMAO concentration, which was observed in the large-scale studies in younger populations^9,29^. Finally, due to the retrospective nature of the present study, certain confounding factors affecting the TMAO concentration, such as the change of gut microbiota, permeability of the gut-blood barrier, and long-term dietary habits, could not be fully accessed.

In conclusion, plasma TMAO increased intracellular oxidative stress and inflammatory cytokines, contributed to impaired functions as well as reduced NO production in human EPCs. An elevated TMAO concentration was associated with fewer circulating EPCs, a lower FMD value, and a higher incidence of MACEs in patients with angina pectoris. These findings provide evidence of the potential toxicity of TMAO on EPCs and deliver new insight into the mechanism of atherosclerosis regulation by intestinal microbiota.

## Methods

### Study population

**Figure 4:**
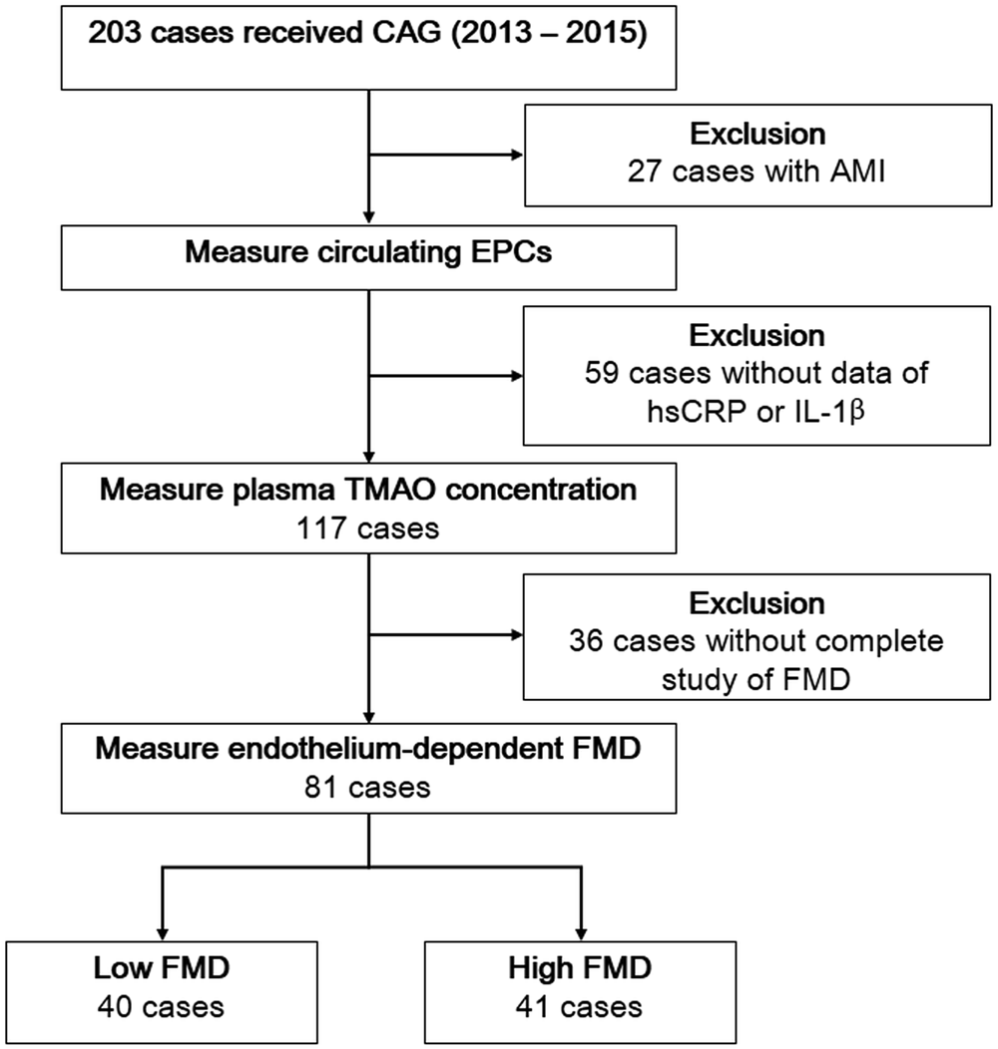
Flowchart of patient enrollment. AMI, acute myocardial infarction; CAG, coronary angiography; EPC, endothelial progenitor cells; FMD, flow-mediated vasodilation; hsCRP, high-sensitivity C-reactive protein; IL-1β, interkeukin-1β; TMAO, trimethylamine N-oxide.

We retrospectively screen 203 patients who was more than 18 years of age, with stable angina, and admitted for cardiac catheterization at Taipei Veterans General Hospital from January 2013 to April 2015. After excluding patients with acute myocardial infarction (n = 27), absence of pre-procedure hsCRP and IL-1β data (n = 59), or incomplete study of FMD (n = 36), 81 patients were enrolled for analysis. Flowchart of patient enrollment was summarized in Fig. 4. Data regarding comorbidities, smoking status, and pre-procedure laboratory exams were collected from each patient. The blood cell count, plasma creatinine, glucose, lipid profiles, and uric acid were measured by routine laboratory methods. The estimated glomerular filtration rate (eGFR) was calculated by the Modification of Diet in Renal Disease equation for Japanese subjects^30^. Coronary angiography (CAG) and left ventriculography (LVG) were performed for all enrolled subjects. A coronary lesion with narrowing of >50% in diameter was defined as significant stenosis. Patients with significant stenosis in at least one vessel were suggested to have significant CAD. Based on the CAG results, SYNTAX scores^31^ were calculated (http://www.Syntaxscore.com). The left ventricular ejection fraction (LVEF) was estimated by LVG, and the left ventricular end-diastolic pressure (LVEDP) was measured with a pigtail catheter placed in the mid-left ventricular cavity. This research was conducted according to the principles expressed in the Declaration of Helsinki. All participants provided their written informed consents, and the study was approved by the Research Ethics Committee of Taipei Veterans General Hospital.

### Measurement of TMAO and inflammatory markers

For each patient, 20 mL of blood was obtained to measure the TMAO and inflammatory markers after fasting and cessation of medications for at least 12 hours. After the blood samples were centrifuged, all plasma samples were stored at −20 °C until the measurement of TMAO and other inflammatory cytokines in the batched assays, which occurred approximately 1 week later. For each case, 200 μL of plasma was mixed with TMAO-d9 isotopologues. After filtration, plasma TMAO was quantified with the use of a stable isotope dilution assay and high-performance liquid chromatography with online electrospray ionization tandem mass spectrometry on an API 4000 Q-TRAP mass spectrometer (AB SCIEX, Framingham, MA, USA). The concentrations of high-sensitivity C-reactive protein (hsCRP) were assessed using a commercial enzyme-linked immunosorbent assay (ELISA; Beckman Coulter Inc., Brea, CA, USA); and the concentrations of interkeukin-1β (IL-1β) were assessed using the human IL-1β/IL-1F2 Immunoassay (R&D Inc., Minneapolis, MN, USA).

### Measurement of circulating EPCs

Quantification of the circulating EPCs by flow cytometry was performed as described previously^32^. The peripheral blood of patients was incubated with human kinase insert domain receptor (KDR) antibody (R&D, Minneapolis, MN, USA) for 30 min in the dark, and then by allophycocyanin-conjugated secondary antibody, phycoerythrin-conjugated human CD133 antibody (Miltenyi Biotec, Germany), and FITC-conjugated human CD34 antibody (Becton Dickinson Pharmingen, USA). After incubation for 30 min, the cells were washed with phosphate-buffered saline before analysis. Each analysis included 100,000 events. We measured the numbers of circulating EPCs gated with monocytes, which were defined as CD34^+^KDR^+^ or CD34^+^KDR^+^CD133^+^. Reproducibility of the EPC measurements has been confirmed in our previously published studies^33^, in which we measured the circulating EPCs from two separate blood samples from 10 subjects.

### Endothelium-dependent flow-mediated vasodilation (FMD) and Clinical Endpoints

FMD was assessed using a 7.5-MHz linear array transducer (Hewlett-Packard Sonos 5500, Andover, MA, USA) to scan the brachial artery in the longitudinal section. The FMD measurement was performed as described previously^15^. A baseline image was acquired, and blood flow was estimated by time-averaging the pulsed Doppler velocity signals obtained from a mid-artery sample volume. The cuff was inflated to at least 50 mmHg above systolic pressure to occlude the brachial artery for 5 minutes. A mid-artery-pulsed Doppler signal was obtained immediately upon cuff release to assess the hyperemic velocity. Post-occlusion diameters were obtained at 60, 100, and 120 seconds after deflation. Endothelium-dependent FMD was calculated as the maximal post-occlusion diameter relative to the averaged pre-occlusion diameter. Subjects were classified according to the FMD results. Subjects with FMD values greater than the median were classified as the “high-FMD group;” otherwise, they were classified as the “low-FMD group.” The enrolled subjects were followed-up until December 2016 or until the occurrence of a major adverse cardiovascular event (MACE), including target vessel revascularization, nonfatal myocardial infarction (MI), and death. Target vessel revascularization was defined as balloon dilatation or stent deployment over a previously treated lesion. Nonfatal myocardial infarction was defined as elevation of cardiac troponin I (>1 ng/mL) with ischemic symptoms. All patients received regular follow-up appointments at our Out-Patient Department, and their medical records were carefully reviewed.

### *In vitro* studies of EPCs

Peripheral blood samples for EPC culture were obtained from healthy young volunteers. Details of the culture of EPCs were provided in the Supplement. In brief, EPCs were seeded at approximately 1 × 10^4^ cells on 24-well plates. After reaching 70% confluency, the cells were treated with different concentration (0, 2, 100, 200, and 500 μM) of TMAO for 24 hours. TMAO was purchased from Sigma-Aldrich, dissolved in distilled water, and then diluted in the culture medium for different concentrations to be used in the study. The cell viability of EPCs was determined by 3-(4,5-dimethylthiazol-2-yl)−2,5,diphenyltetrazolium bromide (MTT) assay. Human EPCs were incubated with MTT (0.5 mg/ml, Sigma) for 4 hours, lysed with dimethyl sulfoxide, and their absorbance were measured by a microplate reader at 550/650 nm.

After the cells had been treated with different concentrations of TMAO for 24 hours, the medium of culture was then collected for cytokine measurement. The concentrations of IL-6, CRP, and tumor necrosis factor-α (TNF-α) in the medium were determined using ELISA (Sigma-Aldrich), which was performed according to the manufacturer’s protocol. To detect the intracellular H_2_O_2_ and oxidative stress, the EPCs were treated with the 2′,7′- dichlorodihydrofluorescein diacetate probe (DCFH-DA 20 μM, Molecular Probes) for 1 hour. The fluorescence intensity was measured at the excitation wavelength of 485 nm and the emission wavelength of 530 nm by an Infinite® 200 plate reader (TECAN). The intracellular nitric oxide (NO) release from EPCs was quantified using the 4-amino-5- methylamino-2′,7′-difluorofluorescein diacetate (DAF-FM) (Life Technologies). Fluorescence excitation and emission maxima were 495 nm and 515 nm, respectively. The results of ROS/NO production were expressed as the percentage of their fluorescence intensity compared to controls (%).

To access the function of EPC, cell tube formation was performed with an *in vitro* Angiogenesis Assay Kit (Chemicon). ECMatrix gel was mixed with ECMatrix diluent buffer and placed in a 96-well plate at 37 °C for 1 hour to allow the matrix solution to solidify. Totally 1 × 10^4^ EPCs were placed on a matrix solution with medium and incubated at 37 °C for 16 hours. Tubule formation was inspected under an inverted light microscope (×100). Six random fields were taken and counted by using computer software, Image-Pro Plus. In addition, the migratory function of EPC was evaluated by a modified Boyden chamber (Transwell, Costar). In the lower chambers, stromal cell-derived factor 1 (SDF-1) (50 ng/mL) was supplemented to the medium and 4 × 104 EPCs were placed in the upper chambers of transwell plates with serum-free endothelial growth medium placed at 37 °C incubation for 24 hours. The membrane of chamber was washed by phosphate buffered saline (PBS) twice and stained using hematoxylin (Merck Millipore). Then the upper membrane side was scraped with a cotton ball and fixed with 2% parafor-maldehyde. The migrated cells in lower membrane side were counted by 8 random high-power (×100) microscopic fields by light microscopy.

### Statistical analysis

Data were expressed as the median (quartiles) for numeric variables and as a number (percent) for categorical variables. Clinical and laboratory data were compared using the Mann-Whitney U test for continuous variables and Fisher’s exact test for categorical variables. Spearman’s rank correlation test was used to assess the correlation between TMAO, FMD, and other variables. Survival curves were generated with the Kaplan–Meier method, and survival among groups was compared by the log-rank test. Linear regression analysis was performed to investigate the relationships of various factors and FMD, which was logarithmically (log) transformed to achieve a normal distribution before analysis. Factors with statistical significance by univariate regression analysis were further entered into multivariate regression analysis. Data were analyzed using SPSS version 18.0 (SPSS Inc., Chicago, IL, USA) and MedCalc version 11.4.2.0 (MedCalc Software, Mariakerke, Belgium). A *p*-value less than 0.05 was regarded as statistically significant.

## Supplementary information

Supplement

## Data Availability

The datasets generated during and/or analysed during the current study are available from the corresponding author on reasonable request.
