# Supplementary material for: Trimethylamine N-Oxide, Circulating Endothelial Progenitor Cells, and Endothelial Function in Patients with Stable Angina
Source: Sci Rep. 2019 Mar 12;9:4249. doi: 10.1038/s41598-019-40638-y (PMC6414518; doi:10.1038/s41598-019-40638-y)
Supplement: Supplementary file 1 — Supplement [file 41598_2019_40638_MOESM1_ESM.pdf]

SUPPLEMENT

Trimethylamine N-Oxide, Circulating Endothelial Progenitor Cells, and Endothelial Function in Patients with Stable Angina

Ruey-Hsing Chou[1,3,5,7], Chi-Yu Chen[1], I-Chun Chen[1], Hsin-Lei Huang[2,10], Ya-Wen Lu[3,5], Chin-Sung Kuo[1,3,6,*], Chun-Chin Chang[1,3,5], Po-Hsun Huang[1,3,5,7,*], Jaw-Wen Chen[3,4,5,9], Shing-Jong Lin[3,5,8,11]

[1] Institute of Clinical Medicine, National Yang-Ming University, Taipei, Taiwan.

[2] Institute of Physiology, National Yang-Ming University, Taipei, Taiwan.

[3] Cardiovascular Research Center, National Yang-Ming University, Taipei, Taiwan.

[4] Institute and Department of Pharmacology, National Yang-Ming University, Taipei, Taiwan.

[5] Division of Cardiology, Department of Medicine, Taipei Veterans General Hospital, Taipei, Taiwan.

[6] Division of Endocrinology and Metabolism, Department of Medicine, Taipei Veterans General Hospital, Taipei, Taiwan.

[7] Department of Critical Care Medicine, Taipei Veterans General Hospital, Taipei, Taiwan.

[8] Healthcare and Management Center, Taipei Veterans General Hospital, Taipei, Taiwan.

[9] Department of Medical Research, Taipei Veterans General Hospital, Taipei, Taiwan.

[10] Department of Nursing, National Taipei University of Nursing and Health Sciences.

[11] Taipei Medical University, Taipei, Taiwan.

Methods

Human endothelial progenitor cell (EPC) culture

Peripheral blood samples for EPC culture were obtained from healthy young volunteers. Total mononuclear cells (MNCs) were isolated by density gradient centrifugation with Histopaque-1077 (1.077 g/mL, Sigma) from the peripheral blood of the healthy young volunteers. Briefly, 5×10^6 MNCs were seeded in EGM-2MV medium (Cambrex, East Rutherford, NJ, USA), supplemented with hydrocortisone, human epidermal growth factor, R3-insulin-like growth factor 1, human fibroblast growth factor, vascular endothelial growth factor, gentamicin, amphotericin B, vitamin C, and 20% fetal bovine serum, on 0.1% fibronectin-coated plates. The medium was changed after every 4 days of culture, and EPCs appeared within 7–15 days after the start of the MNC culture. EPC passage numbers 3–7 were used. Additionally, we identified the EPCs by the antibodies CD34, CD133, KDR, CD31 (Santa Cruz), and vWF (Neomarkers) using immunofluorescence assays. The fluorescent images were obtained by a laser scanning confocal microscope.
